# Preparation of Bamboo-Based Hierarchical Porous Carbon Modulated by FeCl_3_ towards Efficient Copper Adsorption

**DOI:** 10.3390/molecules26196014

**Published:** 2021-10-03

**Authors:** Yixin Zhang, Guofeng Qiu, Rumeng Wang, Yang Guo, Fanhui Guo, Jianjun Wu

**Affiliations:** 1 National Engineering Research Center of Coal Preparation and Purification, China University of Mining and Technology, No.1 Daxue Road, Xuzhou 221116, China; 2 Shandong Xuanyuan Scientific Engineering and Industrial Technology Research Institute Co., Ltd., Longgu, Juye, Heze 274918, China; 3 School of Chemical Engineering and Technology, China University of Mining and Technology, No.1 Daxue Road, Xuzhou 221116, China; ts20040045a31@cumt.edu.cn (G.Q.); rumengwang@cumt.edu.cn (R.W.); cumt-guoyang@cumt.edu.cn (Y.G.); cumtgfh@163.com (F.G.)

**Keywords:** bamboo powder, biochar, hierarchical porous carbon, Fe^3+^ ions, adsorption, copper ions

## Abstract

Using bamboo powder biochar as raw material, high-quality meso/microporous controlled hierarchical porous carbon was prepared—through the catalysis of Fe^3+^ ions loading, in addition to a chemical activation method—and then used to adsorb copper ions in an aqueous solution. The preparation process mainly included two steps: load-alkali leaching and chemical activation. The porosity characteristics (specific surface area and mesopore ratio) were controlled by changing the K_2_CO_3_ impregnation ratio, activation temperature, and Fe^3+^ ions loading during the activation process. Additionally, three FBPC samples with different pore structures and characteristics were studied for copper adsorption. The results indicate that the adsorption performance of the bamboo powder biochar FBPC material was greatly affected by the meso/micropore ratio. FBPC _2.5-900-2%_, impregnated at a K_2_CO_3_: biochar ratio of 2.5 and a Fe^3+^: biochar mass ratio of 2%, and activated at 900 °C for 2 h in N_2_ atmosphere, has a very high specific surface area of 1996 m^2^ g^−1^ with a 58.1% mesoporous ratio. Moreover, it exhibits an excellent adsorption capacity of 256 mg g^−1^ and rapid adsorption kinetics for copper ions. The experimental results show that it is feasible to control the hierarchical pore structure of bamboo biochar-derived carbons as a high-performance adsorbent to remove copper ions from water.

## 1. Introduction

Heavy metal pollution of water has been a challenge in many countries around the world for decades and has attracted the attention of researchers due to its serious impact on human health. Because the removal of heavy metals usually requires expensive materials and complex methods, the use of low-cost and environmentally friendly waste forest products is a simple and effective approach. At present, among the heavy metals with toxic effects on humans and the environment, the sources of copper ions can be divided into natural and man-made, such as volcanic activity, mining, and smelting, brass manufacturing, electroplating, oil refining, etc., and exist in the aquatic environment, which grants easy entry into the food chain [1]. According to the World Health Organization, the maximum concentration of copper in drinking water in many countries is 3.0 mg L^−1^ [2]. In the human body, copper is retained by homeostasis [3]. Excessive concentrations of copper ions in the human body can lead to kidney/liver damage as well as Alzheimer’s disease [4]. Many conventional methods, including electrochemical methods, chemical precipitation, chemical coagulation, membrane filtration, ion exchange, and bioremediation, have been used to remove copper from natural water sources and wastewater [5,6,7,8]. However, most of these treatment techniques are environmentally damaging and suffer from lower efficiency, operational limitations, and high cost, all of which restrict their application. Compared with other technologies, adsorption is considered to be the most effective physical and chemical technology to remove heavy metals because of its simple operation, cost-effectiveness, and the regeneration properties of the adsorbent [9]. Many studies on the adsorption of copper by activated carbon and waste biomass adsorbents have been carried out [10,11,12]. It is worth noting that in the adsorption process, the adsorbent is considered to be the most important factor affecting the removal efficiency of pollutants. Therefore, it is very important to find a cost-effective and environmentally friendly adsorbent to remove copper.

In recent years, the application of biomass resources in the preparation of porous carbon materials has attracted much attention because of its renewability. As waste from the paper industry, bamboo powder has the advantage of being economical and widely available. In addition, hierarchical porous carbon has interesting advantages due to its special structure [10,13], so there have been a lot of studies related to it. Interconnections among macropores, mesopores, and micropores within hierarchical porous carbon structures facilitate rapid ion transport [14]. Macropores and mesopores promote rapid ion transfer through channels to micropores [15], resulting in rapid mass transfer kinetics and low mass transfer resistance [12].

Bamboo is composed of cellulose, hemicellulose, and lignin. The carbonization of lignocellulosic components mainly results in the formation of micropores [16]. To obtain the typical char structure mentioned above, Fe-based compounds are used for the following reasons. First, Fe-based compounds (such as Fe_2_O_3_ and FeCl_3_) can be used as templates to synthesize mesoporous materials [17,18]. Mixtures containing biomass carbon and metallic compounds are heated to high temperatures. After pyrolysis, the metal compounds in biomass carbon can be easily removed with diluted hydrochloric acid and a clear mesoporous structure can be obtained. Secondly, iron-based compounds can also improve the microstructure. Xu et al. [19]and Oztas et al. [20] showed that iron-based compounds could catalyze the decomposition of all hydrocarbons in biomass carbon, with the exception of methane. This reaction promotes the formation and release of volatiles and hinders the condensation of free radicals during pyrolysis. Finally, iron is a common natural ingredient, and it has a lower cost than other metals such as vanadium, nickel, and zirconium. Therefore, the preparation of a typical biomass charcoal material in the pyrolysis process can be achieved by changing the amount of iron-based compounds. These factors play an important role in determining the development of pores during activation, but they are rarely discussed systematically.

In this study, using bamboo powder biochar as a raw material and FeCl_3_ and K_2_CO_3_ as additives, the effects of different contents of FeCl_3_ and K_2_CO_3_ on the pore structure (the specific surface area and the V_meso_/V_total_ ratio) and the physical and chemical properties of FBPC samples were studied. In addition, the effects of the adsorption isotherm, kinetics, and initial pH on the adsorption of copper ions on different FBPC materials were studied through batch experiments to determine the adsorption behavior of copper ions on different FBPC materials.

## 2. Results and Discussion

BC was prepared by carbonizing bamboo powder in a process in which the pyrolysis of the organic components of the feedstock included the release of oxygen and hydrogen as carbon monoxide, carbon dioxide, H_2_ and H_2_O, and condensable volatiles to obtain the carbon material [21]. FBPC was then manufactured through supported Fe^3+^ ion catalysis and a chemical activation process to improve the porous structure by eliminating remaining inorganic and amorphous carbon [22].

### 2.1. Physical Characteristics

**Figure 1:**
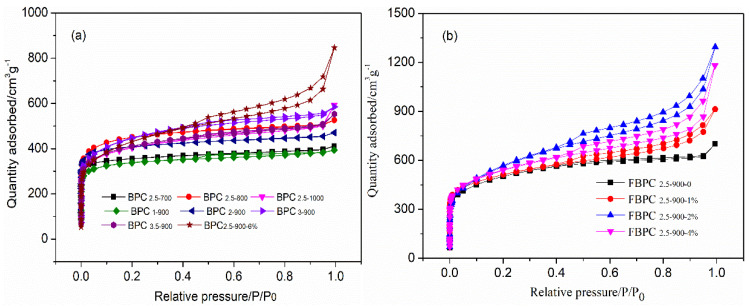

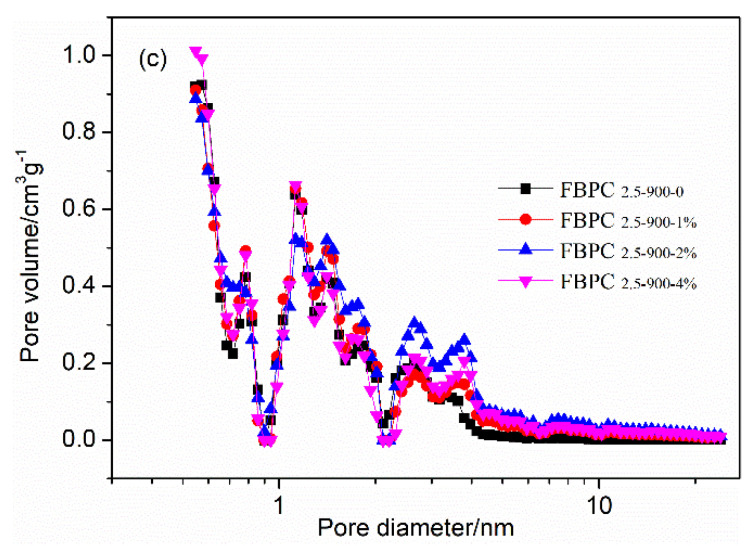
(**a**) Nitrogen adsorption/desorption isotherms of the BPC and FBPC samples; (**b**) nitrogen adsorption/desorption isotherms; and (**c**) DFT pore size distribution of the FBPC samples.

The physical properties of the material were studied to estimate the porosity development within the BC to form a hierarchical porous structure in the FBPC sample. The specific surface area, total pore volume, and pore width of BC, BPC, and FBPC samples were determined by nitrogen sorption and desorption. The nitrogen adsorption–desorption isotherms and pore size distributions are shown in Figure 1a–c. As shown in the figure, the FBPC _2.5-900-1%_, FBPC _2.5-900-2%_, FBPC _2.5-900-4%_, and BPC _2.5-900_ (FBPC _2.5-900-0%_) samples all belonged to type I isotherms and type H4 hysteresis loops, suggesting that micropores and mesopore coexisted [15,22]. The most interesting aspect was the apparent hysteresis of the rings in the FBPC _2.5-900-1%_, FBPC _2.5-900-2%_, and FBPC _2.5-900-4%_ samples, but for BPC _2.5-900_, no such rings were observed. This difference indicated that FBPC _2.5-900-1%_, FBPC _2.5-900-2%_, and FBPC _2.5-900-4%_ samples had a higher number of mesopores. In addition, the density function theoretical model was used to calculate the pore size distribution of FBPC samples. All FBPC samples loaded with Fe^3+^ ions mainly consisted of micropores in the range of 0.6 to 2.0 nm and mesoporous pores in the range of 2.0 to 7 nm. However, the mesoporous range of BPC _2.5-900_ (FBPC _2.5-900-0%_) was mainly 2 to 5 nm.

The results in Table 1 show that the specific surface area of the BC sample was only 468 m^2^ g^−1^ after the carbonization step of the organic compound was removed. By using chemical activation and catalytic processes, the specific surface area of the BPC samples was significantly increased, i.e., it was in the range of 1301 to 1996 m^2^ g^−1^. At the same time, the ratio of mesopore to total pore volume (V_meso_/V_total_) ranged from 0.11 to 0.581. Therefore, FBPC samples with high specific surface area and adjustable mesoporous/microporous ratio can be prepared by controlling three operating parameters during the activation process: the temperature, the K_2_CO_3_ to BC ratio, and amount of FeCl_3_ added.

It was obvious that the activation temperature was one of the key factors for the specific surface area and pore volume of strong BPC samples. The specific surface area of BPC samples increased from 1405 m^2^ g^−1^ of BPC _2.5-700_ to 1793 m^2^ g^−1^ of BPC _2.5-900_, which corresponded to activation temperatures of 700 °C and 900 °C, respectively. Notably, the V_meso_/V_total_ of the BPC _2.5-700_ and BPC _2.5-900_ samples was 0.11 and 0.312, respectively, indicating a significant enhancement of mesopore. The increase in pore structure in the BPC sample was most likely due to the widening of the micropores towards the mesopores [23]. However, when the temperature continued to rise from 900 °C to 1000 °C, the specific area and pore volume decreased (the total specific surface area decreased from 1996 m^2^ g^−1^ to 1528 m^2^ g^−1^, and V_total_ decreased from 0.894 to 0.724), but the pore size increased. This was the case because higher activation temperatures lead to more carbon atoms being involved in the reaction, and larger numbers of etched pores lead to more metal potassium turning into vapor (the boiling point of potassium is 762 °C) and entering the middle of the carbon layer for further etching. This reaction can broaden the radius of the hole, while it will also break the generated pore structure, resulting in the collapse of some pores, and thus, the specific surface area and pore volume of the obtained material will decrease. In addition, the impregnation ratio of K_2_CO_3_: BC affected the generation of new pores, widened existing pores, and controlled the ratio of mesopores to micropores. At 900 °C, the specific surface area increased significantly from 1301 m^2^ g^−1^ in the BPC _1-900_ sample to 1793 m^2^ g^−1^ in the BPC _2.5-900_ sample, and the V_meso_/V_total_ increased from 0.14 to 0.312. However, when the K_2_CO_3_: BPC ratio further increased to 3.5 in the BPC _3.5-900_ sample, the specific surface area gradually decreased, but the V_meso_/V_total_ decreased to 0.263. This reduction may have been due to excess K_2_CO_3_ reacting with carbon atoms to convert carbon dioxide in a gaseous form, with some mesopores collapsing during the activation time period [11,24].

From Figure 1b,c, and Table 1, it can be seen that an increase in Fe^3+^ ions loading from 0% to 2% led to an increase in the specific surface area, mesopore volume, and V_meso_/V_total_. However, for the FBPC _2.5-900-2%_ and FBPC _2.5-900-6%_ samples, the loading of Fe^3+^ ions increased from 2% to 6%, the total specific surface area decreased from 1996 m^2^ g^−1^ to 1537 m^2^ g^−1^, and the V_meso_/V_total_ value decreased from 0.581 to 0.474. From these results, it can be concluded that with the increase in the loading capacity, the adsorption curve was gradually warped upward. From the pore size distribution and pore structure parameters, it can be seen that the micropore volume of different pore sizes (0–2 nm) increased significantly with the increase in the micropore diameter of iron-carrying precursors, and the total pore volume also continued to increase. The pore reaming of the micropores also generated a part of the mesopores, resulting in a rapid increase in the specific surface area. The results show that the presence of iron compounds in the activation process inhibited the growth of aromatic lamellae and their longitudinal polycondensation behavior, promoted the further cracking of large aromatic rings to small aromatic rings and the generation of active sites, weakened the diffusion resistance of activated gases in the particles, and fully etched the microcrystalline structure to generate a large number of new micropores and mesopores [25]. For FBPC _2.5-900-6%_, the aggregation behavior of a relatively small number of catalysts that were dispersed at high temperature during the activation process led to a decrease in catalytic cracking activity and a gradual decrease in the transformation speed of its microscopic carbon structure to the disordered direction, which affected the rapid generation of micropores and mesopores in the particles.

**Figure 2:**
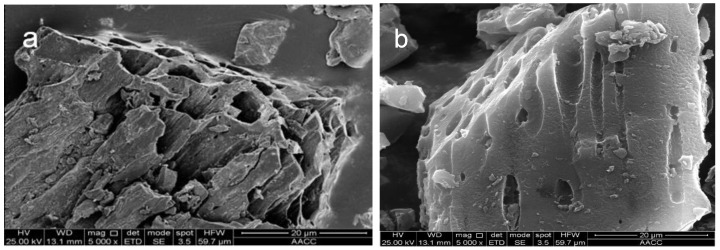

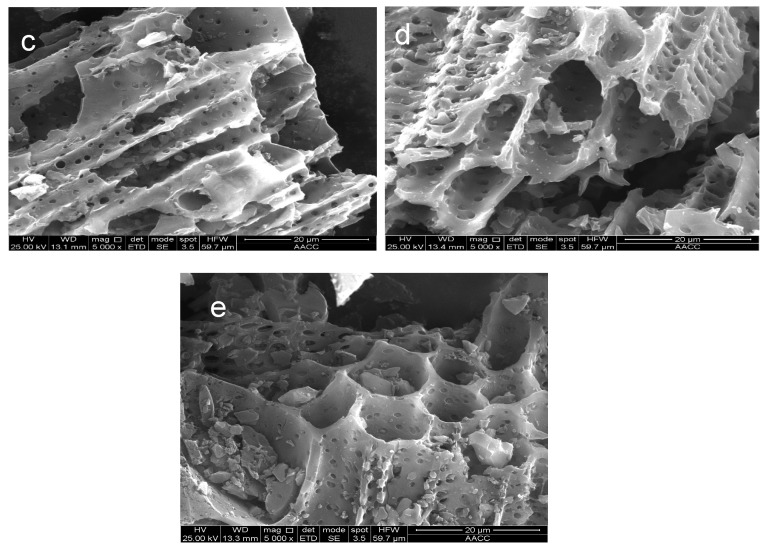
SEM images of (**a**) BC, (**b**) BPC _2.5-900_, (**c**) FBPC _2.5-900-1%_, (**d**) FBPC _2.5-900-2%_ and (**e**) FBPC _2.5-900-4%_ with ×5000 magnification.

The surface morphology and structural properties of the BC, BPC _2.5-900_, FBPC _2.5-900-1%_, FBPC _2.5-900-2%_ and FBPC _2.5-900-4%_ samples were observed by SEM. As shown in Figure 2a, the vascular bundles of bamboo shavings were well preserved after the carbonization process, with macroporous channels ranging in size from 3 μm to 9 μm. After impregnation and activation with the addition of K_2_CO_3_, it could be observed that the sample was in the original macropores and the micropores became more abundant. However, after the chemical activation process, which included loading with Fe^3+^ ions in addition to K_2_CO_3_ impregnation, it could be seen from Figure 2c, d and e that the surface of the sample was rougher and more porous than that of the BC and BPC _2.5-900_ samples, which represented strong evidence of the pore development process. Importantly, there was a change in the inner epidermis surface morphology from a flat and solid surface to a hollow porous structure, as shown in Figure 2a–c, which fully demonstrated the successful preparation of hierarchical porous carbons.

This section provides evidence that the pore characteristics of FBPC can be controlled by the method of loading Fe^3+^ ions and K_2_CO_3_ activators together. It is worth noting that the well-controllable mesoporous structure and high specific surface area of FBPC can ensure the effective removal of copper ions during the adsorption process. In addition, the successful preparation of FBPC samples with a high mesoporous ratio and the high specific surface area also provides a logical basis for the implementation of the following work.

### 2.2. Chemical Characteristics

**Figure 3:**
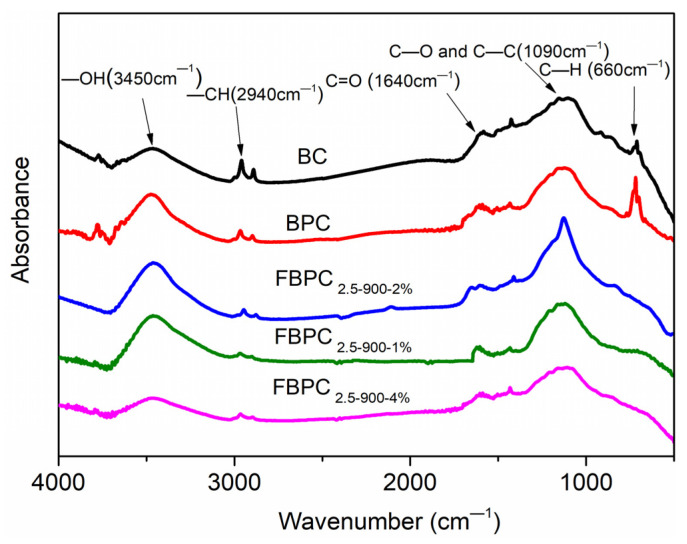
FTIR spectra of the BC, BPC _2.5-900_, FBPC _2.5-900-1%_, FBPC _2.5-900-2%_ and FBPC _2.5-900-2%_ samples.

The chemical properties of the adsorbent were studied to understand and explain some of the adsorption phenomena reported in the next section. FTIR analysis is an important means to identify the surface functional groups of adsorbents, which drive the adsorption of heavy metal ions through the chemical mechanism. The characteristic bands of the functional groups of the BC, BPC, and FBPC samples are presented in Figure 3. The wide band of the samples at around 3450 cm^−1^ is ascribed to the O-H stretching mode of hydroxyl groups or adsorbed water. The band observed near 2950 cm^−1^ is attributed to the C-H stretching vibration of -CH_2_. With the increase in the preparation temperature, the relative strength of the band decreased, indicating the loss of -CH_2_ functional groups in carbon prepared at higher temperatures [26]. A small band in the range of 1500 to 1700 cm^−1^ was found in the sample, which is attributed to the C=O stretching vibration of the ketone, aldehyde, lactone, and carboxyl groups [27,28]. Due to the stretching vibration of the C-O and C-C bond, a band was detected at about 1100 cm^−1^ in all samples [29]. In addition, the peaks in the range of 900–650 cm^−1^ represent aromatic C-H bending vibrations [4,30]. The analysis of biochar materials loaded with Fe^3+^ ions showed that the peak at 660cm^−1^ in biochar materials without Fe^3+^ ions was repressed, suggesting that the surface modification concealed this functionality [31].

**Figure 4:**
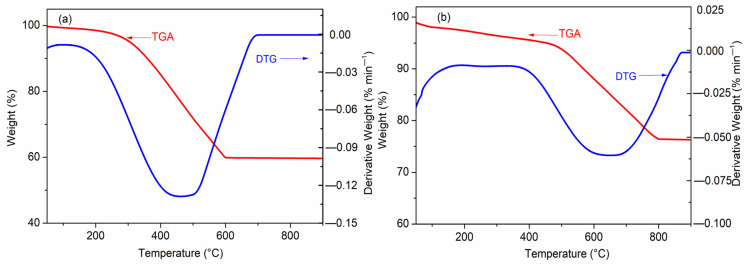
TGA-DTG thermograms for the (**a**) BC and (**b**) FBPC _2.5-900-2%_ samples under an N_2_ flow that was similar to that of the activation process.

In order to further analyze the thermal stability and composition of BC and FBPC _2.5-900-2%_, Figure 4 shows the thermogravimetric analysis (TGA) results for the BC and FBPC _2.5-900-2%_ samples in the temperature range from 50 °C to 900 °C, at a fixed heating rate of 10 °C min^−1^, under nitrogen flow. The pyrolysis process of the BC sample was divided into two stages. The early weightlessness of about 4.6% of the BC, observed at around 310 °C, was due to the loss of water and volatile organic compounds in the microporous structure of the BC [32]. Subsequently, the weight loss curve of the BC sample showed a rapid weight loss of about 35.67% between 310 and 600 °C, which was mainly caused by carbon dioxide being released due to the gradual decomposition of biopolymer components (such as hemicellulose, cellulose, and lignin) [33].

Similarly, the early weight loss of about 6.2% of the FBPC _2.5-900-2%_ was observed at 460 °C due to the presence of water molecules and volatile substances. The early weight loss of FBPC _2.5-900-2%_ was higher than that of the BPC samples, which was caused by the higher water storage of the highly porous structure of the FBPC _2.5-900-2%_ samples. In addition, compared with the BC sample, the weight loss of the FBPC _2.5-900-2%_ sample in the temperature range of 460 to 810 °C was only 26% during the rapid weight loss phase, because the supported iron-based compounds could catalyze the decomposition of all hydrocarbons in biomass carbon and promote the formation and release of volatiles. Therefore, the weight loss of FBPC _2.5-900-2%_ was small, which provides further evidence that the thermal stability of FBPC _2.5-900-2%_ is better than BC.

The research results in this section show that FBPC samples have strong chemical properties and rich functional groups (OH, C-H, C=O, COOH, C-C, and C-O), as well as good thermal stability. These properties can greatly improve the adsorption performance of Cu(II) ions in water.

### 2.3. Adsorption Studies

**Figure 5:**
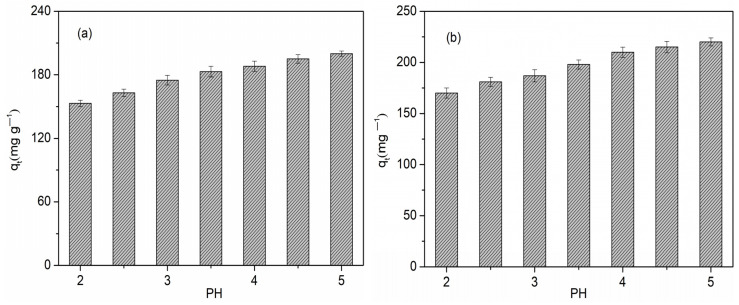

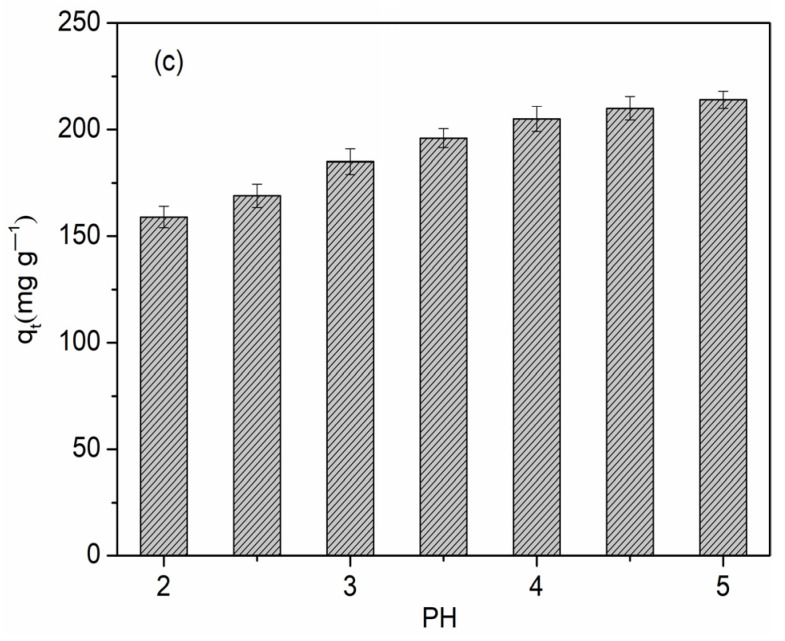
Effect of the initial pH value on Cu(II) adsorption on (**a**) FBPC _2.5-900-1%_, (**b**) FBPC _2.5-900-2%_, (**c**) FBPC _2.5-900-4%_. Adsorbent dose = 0.35 g L^−1^; shaking time = 120 min; temperature = 298 K; pH = 2–5; shaking speed = 300 rpm; initial Cu(II) concentration = 100 mg L^−1^.

According to the characteristics of porosity, three samples of FBPC _2.5-900-1%_, FBPC _2.5-900-2%_, and FBPC _2.5-900-4%_ were selected to evaluate the influence of pore properties on Cu(II) adsorption. It can be seen from Table 1 that compared with the other two FBPC samples, FBPC _2.5-900-2%_ had the highest specific surface area (1996 m^2^g^−1^) and the highest mesoporous ratio (V_meso_/V_total_ = 0.581). The experiment on Cu(II) adsorption was carried out in the initial pH range of 2 to 5. Copper could precipitate when the pH was higher than 5, which would interfere with the measurement of Cu(II) in the adsorption process [26,34]. As can be seen from Figure 5, differences in the initial pH values had a significant influence on the adsorption of Cu(II) ions to FBPC _2.5-900-1%_, FBPC _2.5-900-2%_, and FBPC _2.5-900-4%_. Notably, at the lower initial pH values of 2 and 2.5, the adsorption of copper was significantly lower than at the higher pH values. For example, the adsorption capacity of Cu(II) on FBPC _2.5-900-2%_ increased from 165 mg g^−1^at pH 2 to 220 mg g^−1^ at pH 5. This occurred because the affinity between Cu(II) ions and the adsorbed surface was reduced. In addition, at lower pH values, the competition between H_3_O^+^ and Cu(II) at exchange sites occurred on the adsorption surface [35]. Additionally, the high solubility and ionization of copper salts in acidic media also led to reduced copper removal efficiency. In addition, as the pH value increased from 3 to 5, the amount of Cu(II) adsorbed on the FBPC adsorbent also increased. This improvement could be attributed to the partial hydrolysis of Cu(II) ions, resulting in the formation of Cu(OH)^+^. As previously reported [36,37], CuOH^+^ was more adsorptive than Cu^2+^. More importantly, a possible reason was that when the pH value was higher than 3–4, the hydroxyl (OH) and carboxyl (COOH) groups on the FBPC surface were deprotonated and negatively charged, thereby increasing the attraction of the sample surface to positively charged Cu(II) ions [34,38].

**Figure 6:**
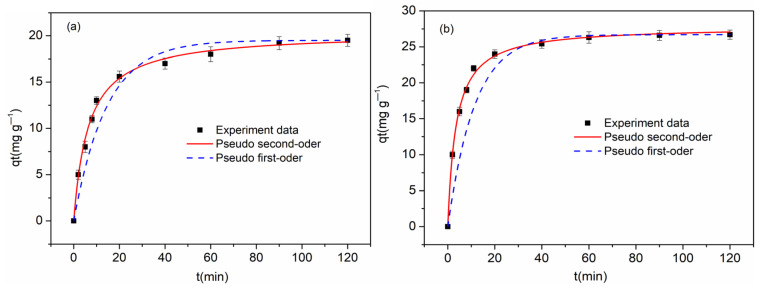

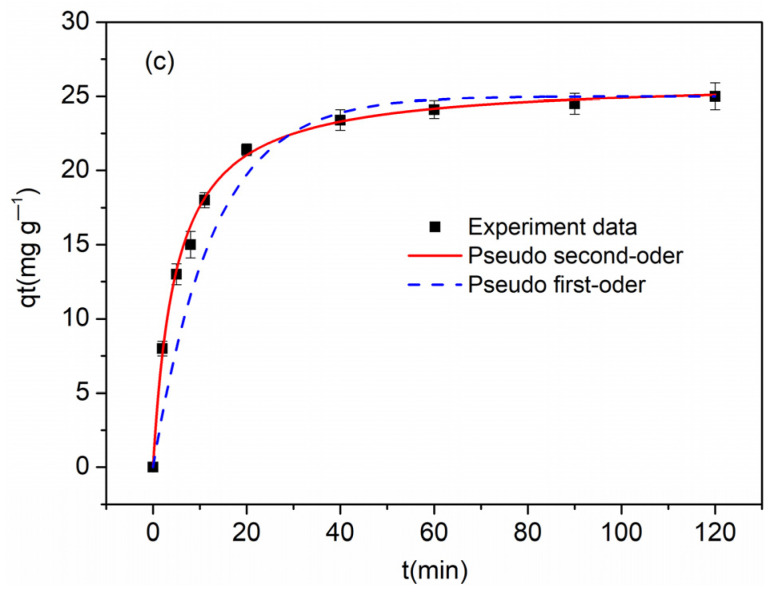
Kinetics of Cu(II) adsorption on (**a**) FBPC _2.5-900-1%_, (**b**) FBPC _2.5-900-2%_, and (**c**) FBPC _2.5-900-4%_. Adsorbent dose = 0.35 g L^−1^; shaking speed = 300 rpm; shaking time = 5–120 min; initial Cu(II) concentration = 10 mg L^−1^; pH = 5; and temperature = 298 K.

The adsorption kinetics of Cu(II) ions on three samples, FBPC _2.5-900-1%_, FBPC _2.5-900-2%_, and FBPC _2.5-900-4%_, are shown in Figure 6a–c and Table 2. As shown, Cu(II) adsorption exhibited an initial extremely fast adsorption time, with 80% of the total adsorption occurring within 25 min of the contact time. Then, the amount of copper adsorbed on the three FBPC samples increased slightly before equilibrium was reached. The fast adsorption of the three high-performance carbon adsorbents could be explained by their hierarchical porous structure. It is worth noting that the Cu(II) adsorption process of the FBPC _2.5-900-2%_ sample reached equilibrium after 70 min, which was much shorter than the adsorption process (100 min) of the FBPC _2.5-900-1%_ and FBPC _2.5-900-4%_ samples.

**Figure 7:**
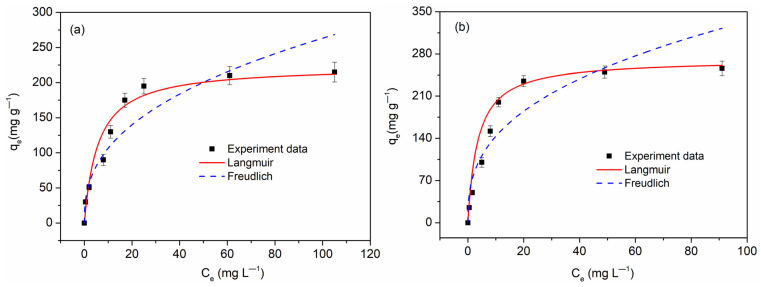

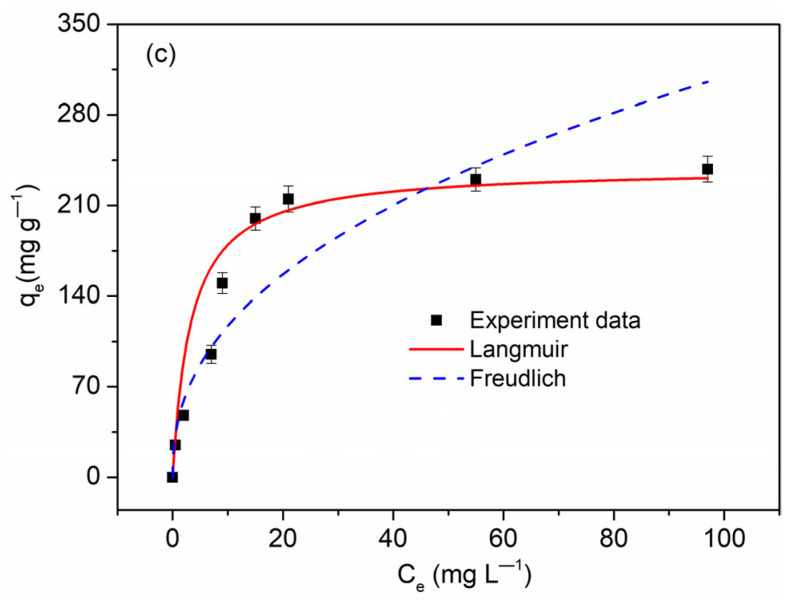
Isotherm of Cu(II) adsorption on (**a**) FBPC _2.5-900-1%_, (**b**) FBPC _2.5-900-2%_, and (**c**) FBPC _2.5-900-4%_. Adsorbent dose = 0.35 g L^−1^; shaking time = 90 min; temperature= 298 K; pH = 5.0; shaking speed = 300 rpm; and initial Cu(II) concentration = 10–200 mg L^−1^.

The adsorption behavior of Cu(II) on the FBPC samples was simulated by using pseudo-first-order and pseudo-second-order nonlinear equations to fit the kinetic data. The mathematical equations of these two models are given in Equations (1) and (2), respectively [39].

(1) $$q_{t}=q_{e}\left(1-e^{-K_{1}t}\right)$$

(2) $$q_{t}=\frac{q_{e}^{2}K_{2}t}{1+q_{e}K_{2}t}$$

where *q_e_* and *q_t_* are the amounts of copper ions adsorbed (mg g^−1^) on the adsorbent at the equilibrium and at time t, respectively, *K_1_* (L h^−1^) is the rate constant of the pseudo-first-order adsorption, and *K_2_* (g mg^−1^ h^−1^) represents the pseudo-second-order adsorption. The kinetic parameters obtained using the pseudo-first-order and pseudo-second-order nonlinear equation fitting are listed in Table 2. It is clear from the above table that the pseudo-first-order equation had a poor fitting degree with the experimental data. On the contrary, the pseudo-second-order model had a better fitting effect, and the fitting coefficient could reach more than 99%. This reflects the fact that the adsorption of Cu(II) on the three FBPC samples was mainly a chemical mechanism [30,34,40]. The k_2_ value of FBPC _2.5-900-2%_ was 0.011g mg^−1^ h^−1^, which was significantly higher than those of the FBPC _2.5-900-1%_ and FBPC _2.5-900-4%_ samples (0.007 and 0.009g mg^−1^ h^−1^, respectively). Thus, it could be concluded that the FBPC _2.5-900-2%_ samples, with their high V_meso_/V_total_ ratios, reached adsorption equilibrium more quickly than the FBPC _2.5-900-1%_ and FBPC _2.5-900-4%_ samples. This indicates that a sample having a high mesoporosity ratio causes Cu(II) to be adsorbed more quickly on the sample. The existence of mesopores can provide barrier-free channels for ions to enter the micropores and achieve adsorption on the active sites so that the ions can be quickly transported from the macropores to the active sites in the micropores. In addition, this article also provides an important theoretical basis for studying the adsorption of copper ions on FBPC _2.5-900-1%_, FBPC _2.5-900-2%_, and FBPC _2.5-900-4%_ samples. The experimental results were further fitted with the Langmuir and Freundlich isotherms based on the nonlinear Equations (3) and (4) [11].

(3) $$q_{e}\;=\frac{q_{m}K_{L}C_{e}}{1+K_{L}C_{e}}$$

(4) $$q_{e}=K_{F}C_{e}^{\frac{1}{n}}$$

where *q_m_* (mg g^−1^) is the maximum adsorption capacity, *K_L_* (L mg^−1^) and *K_F_* (mg g^−1^) are constants for the Langmuir and Freundlich models, respectively, n is the Freundlich constant related to adsorption intensity, and *q_e_* (mg g^−1^) is the equilibrium adsorption capacity. The adsorption isotherms and parameters of the three FBPC samples are shown in Figure 7a–c and Table 3. As can be seen from the figure, the Langmuir model fits the results better than the Freundlich model and produces a higher correlation coefficient (R^2^ > 96%). The results show that the monolayer adsorption of Cu(II) occurred on the homogeneous surface of FBPC samples. From the parameters in Table 3, we can see that the three FBPC samples all showed good Cu(II) ion adsorption capacity, among which the FBPC _2.5-900-2%_ sample had the largest adsorption capacity, up to 256 mg g^−1^, which was much higher than those of the other two FPBC samples. Therefore, it can be concluded that the higher specific surface area and mesoporous ratios play an important role in the adsorption of Cu(II) ions. The reasons can be listed as follows: firstly, a higher specific surface area can provide more adsorption sites, and secondly, a higher ratio of mesopores can provide barrier-free channels for ions to enter the micropores and achieve adsorption on the active sites, as well as accelerating the transfer of ions from macropores to the velocity of deep active sites in the micropores.

In previous studies [41], bamboo-based activated carbon was prepared by unloading Fe ions and directly using K_2_CO_3_ chemical activation steps, and a material with a specific surface area of 1264 m^2^ g^−1^ was obtained. The material prepared by loading Fe^3+^ ions prior to activation in this study has a larger specific surface area than the material obtained by Zhang et al. [41]. This shows that the loading of metal Fe^3+^ ions has an important effect on improving the specific surface area and mesopore ratio of bamboo-based activated carbon. The observed Cu(II) adsorption capacity is compared with those of other activated carbon adsorbents prepared from biomass sources described in the literature in Table 4. As can be seen, the FBPC samples prepared in this paper have much higher adsorption capacities and faster adsorption kinetics than other adsorbents. Due to its unique properties (such as high specific surface area, hierarchical porous structure, and high ratio of mesopore to total pore volume), FBPC samples can achieve high-performance adsorption of Cu(II) ions. Therefore, biomass-activated carbon prepared through the loading of Fe^3+^ ions to regulate the pore structure can become a promising copper ion adsorbent in the process of chemical activation.

## 3. Materials and Methods

### 3.1. Preparation of Bamboo Powder Biochar and BPC

Bamboo powder was collected from the Sichuan bamboo chopsticks processing plant (Sichuan Province in China) and washed with distilled water several times to remove dust and some impurities. The cleaned bamboo powder was dried at 105 °C for 24 h, and then fully dried for subsequent use. Then, the bamboo powder was pyrolyzed under nitrogen atmosphere at 600 °C for 2 h to prepare bamboo powder biochar (denoted as BC). BC was impregnated with Potassium Carbonate at different K_2_CO_3_: BC mass ratios of 1, 2, 2.5, 3, or 3.5 and dried at 105 °C in an electrical oven (denoted BPC). Next, it was activated at 700 °C, 800 °C, 900 °C and 1000 °C (heating rate of 5 °C min^−1^) for 2 h in an N_2_ atmosphere. Then, it was neutralized with a 1 M hydrochloric acid aqueous solution and filtered and washed several times with distilled water until the filtrate pH was 7. Finally, BPC was collected after drying at 105 °C for 24 h. The BPC samples, prepared under different conditions, are recorded as BPC _X-Y_, where X is the ratio of K_2_CO_3_ to BPC and Y(°C) is the temperature.

### 3.2. Preparation of Hierarchical Porous Carbon Catalyzed by Fe^3+^ Ions

BC was impregnated at K_2_CO_3_:BC mass ratio 2.5, and then 1%, 2%, 4%, 6%, FeCl_3_ was added (the mass ratio of FeCl_3_:BC was 1%, 2%, 4%, 6%); it was then impregnated through stirring at room temperature for 12 h, and dried in an electric oven at 105 °C. In the N_2_ atmosphere, it was activated at 900 °C for 2 h. Then, the powder was neutralized with a 1 M hydrochloric acid aqueous solution, and then filtered and washed several times with distilled water until the filtrate pH was 7. Finally, FBPC was collected after drying at 105 °C for 24 h. The FBPC samples, prepared under different conditions, are recorded as FBPC _2.5-900-Z_, where Z is the amount of FeCl_3_ added.

### 3.3. Material Characterization

The specific surface area and pore size distribution of the FBPC samples were characterized using N_2_ adsorption–desorption isotherms at 77 K using a Physical adsorption instrument (Quantachrome, autosorb-iQ-2MP, FL, USA); the specific surface area (S_BET_) was calculated using the Brunauer–Emmett–Teller (BET) equation. The density functional theory (DFT) was used to analyze the pore size distribution of the carbon materials. The morphology of the adsorbent was characterized using emission scanning electron microscopy (FE-SEM, S-4800). Using a thermogravimetric (TG) analyzer (PerkinElmer, STA 8000, Waltham, MA, USA) under an N_2_ atmosphere, the thermal stability of the preparation of FBPC samples was evaluated. Fourier Transform Infrared Spectroscopy (FT-IR), in the range of 4000–400 cm^−1^(Nicolet iS10, Thermo Scientific (Waltham, MA, USA)), was used to study the functional groups on the FBPC surface.

### 3.4. Adsorption Experiments

The adsorption of copper ions onto FBPC samples was investigated. All batch experiments were carried out in a 50 mL centrifuge tube with 30 mL solution at a speed of 300 rpm at 298 K. The Cu(II) solutions were prepared by dissolving CuSO_4_·5H_2_O in distilled water. Kinetic studies were conducted with a 10 mg L^−1^Cu(II) solution (pH = 5) and 10 mg FBPC, and samples were collected at time intervals from 5 min to 120 min. For adsorption isotherms, different initial Cu(II) ion concentrations (10–200 mg L^−1^) were agitated with 0.35 g L^−1^ dosages of adsorbent in a shaker at 300 rpm for 90 min. Because the copper began to precipitate at pH values of above 6, the effect of the initial solution of pH was conducted with the pH range of 2 to 5. The pH values were adjusted by 0.1 M HCl and 0.1 M NaOH. After reaching equilibrium, the suspension was filtered with a 0.45 μm membrane to obtain the required supernatant. The equilibrium concentration of heavy metal ions in the supernatant was measured using atomic absorption spectroscopy. The adsorptive capacity of Cu(II) was calculated using Equation (5) as follows:

(5) $$q_{e}=\frac{\left(C_{0}-C_{e}\right)V}{W}\;$$

where *C_0_* (mg L^−1^) and *C_e_* (mg L^−1^) are the initial and equilibrium adsorbate concentrations in the solution, *q_e_* (mg L^−1^) is the adsorption amount of Cu(II) at equilibrium, *W* is the mass of the FBPC sample (g), and *V* is the volume of the solution (L).

## 4. Conclusions

In this study, a series of FBPC samples with high specific surface area and high mesoporous ratio were successfully synthesized using BC as raw material through Fe^3+^ ion-supported catalysis and a chemical activation process. These steps are essential to achieve efficient adsorption of Cu(II) ions. By adjusting different K_2_CO_3_ impregnation ratios, as well as the activation temperature and the Fe^3+^ ion loading, the specific surface area, total pore volume and mesoporosity of the catalyst can be well controlled. The results show that the specific surface area of FBPC _2.5-900-2%_ is 1996 m^2^g^−1^, and the mesoporous ratio is 58.1%. The most obvious finding of this study is that FBPC samples synthesized from activated biochar can obtain high adsorption capacity and rapid removal of copper ions. The large specific surface area can ensure a high adsorption capacity, and the mesopores are more conducive to faster ion removal during the adsorption process. Among them, the maximum adsorption capacity of FBPC _2.5-900-2%_ is 256 mg g^−1^, and its good pore characteristics (hierarchical porous structure, with high V_meso_/V_total_ ratio) are better than the values given in the literature. The research results show that the high specific surface area and high mesoporous structure of bamboo powder biochar are beneficial in terms of achieving high copper adsorption performance.

## Figures and Tables

**Table 1:** Summary of the pore characterization data of the BC, BPC, and FBPC samples.

Adsorbent	S_BET_^a^ (m^2^ g^−1^)	V_total_^b^ (cm^3^ g^−1^)	V_micro_^c^ (cm^3^ g^−1^)	V_meso_^d^ (cm^3^ g^−1^)		V_micro_/V_total_ %	V_meso_/V_total_%	D_ave_(nm)
BC	468	0.303	0.154		0.149	50.8	49.2	2.921
BPC _2.5-700_	1405	0.582	0.518		0.064	89.0	11.0	1.913
BPC _2.5-800_	1688	0.729	0.626		0.103	85.9	14.1	2.130
BPC _2.5-900_	1793	0.894	0.615		0.279	68.8	31.2	2.414
BPC _2.5-1000_	1528	0.724	0.532		0.192	73.4	26.6	2.435
BPC _1-900_	1301	0.556	0.478		0.078	86.0	14.0	1.872
BPC _2-900_	1607	0.760	0.588		0.172	77.3	22.7	2.232
BPC _3-900_	1612	0.798	0.569		0.229	71.3	28.7	2.246
BPC _3.5-900_	1508	0.729	0.537		0.192	73.7	26.3	2.268
FBPC _2.5-900-1%_	1823	1.265	0.608		0.657	48.1	51.9	3.685
FBPC _2.5-900-2%_	1996	1.571	0.659		0.912	41.9	58.1	3.890
FBPC _2.5-900-4%_	1894	1.380	0.618		0.762	44.8	55.2	3.792
FBPC _2.5-900-6%_	1537	0.935	0.492		0.443	52.6	47.4	3.603

^a^ BET—specific surface area. ^b^ total volume of pores. ^c^ volume of micropores. ^d^ volume of mesopores.

**Table 2:** Isotherm adsorption parameters for the process of Cu(II) removal by the FBPC samples.

Sample	*q_e_*_, exp_ (mg g^−1^)	Pseudo-First-Order Equation			Pseudo-Second-Order Equation		
		*q_e_*, _cal_ (mg g ^−1^)	*K_1_* (L h^−1^)	R^2^	*q_e_*_, cal_ (mg g ^−1^)	*K*_2_ (g mg^−1^ h^−1^)	R^2^
FBPC _2.5-900-1%_	19.90	19.07	0.060	0.9612	20.32	0.007	0.9987
FBPC _2.5-900-2%_	27.50	26.70	0.081	0.9425	27.86	0.011	0.9994
FBPC _2.5-900-4%_	25.36	25.01	0.074	0.9718	26.10	0.009	0.9995

Note: *q_e_*_,cal_: calculated uptake capacity; *q_e_*_,exp_: experimental uptake capacity; *K*: rate constant; R^2^: correlation coefficient.

**Table 3:** Kinetic adsorption parameters for the process of Cu(II) removal by the FBPC samples.

Sample	Langmuir			Freundlich		
	*q_m_* (mg g^−1^)	*K_L_* (L g^−1^)	R^2^	*K_F_* (mg g^−1^)	n (L g^−1^)	R^2^
FBPC _2.5-900-1%_	221	0.21	0.96	43.15	2.54	0.87
FBPC _2.5-900-2%_	256	0.30	0.97	61.53	2.72	0.91
FBPC _2.5-900-4%_	238	0.27	0.96	44.25	2.36	0.89

**Table 4:** Comparison of maximum adsorption properties of different adsorbents for Cu(II).

Adsorbent	S_BET_ (m^2^ g^−1^)	V_total_ (cm^3^ g^−1^)	Functional Group	Maximum Capacity (mg g^−1^)	Equilibrium Time (min)	Reference
Chestnut shell-activated carbon	1319	0.57	-OH, C-H, C=C, C=O	98	120	[42]
Grape bagasse-activated carbon	1455	0.88	-OH, C-H, C=C, C=O	44	180	[34]
Rice husk-activated carbons	232	0.15	-OH, C-H, -CH2, C=O, C=C	21	1440	[26]
Hazelnut shell-activated carbon	1651	1.38	—	239	120	[43]
Bamboo shaving-activated carbon	1996	1.571	-OH, C-H, C-C, C=O, C-O	256	70	This work

## Data Availability

Not applicable.

## Sample Availability

Samples of the compounds are available from the authors.
